# Supplementary figures and images for: Protective Effect of Polysaccharides Extracted from Cudrania tricuspidata Fruit against Cisplatin-Induced Cytotoxicity in Macrophages and a Mouse Model
Source: Int J Mol Sci. 2021 Jul 13;22(14):7512. doi: 10.3390/ijms22147512 (PMC8304288; doi:10.3390/ijms22147512)

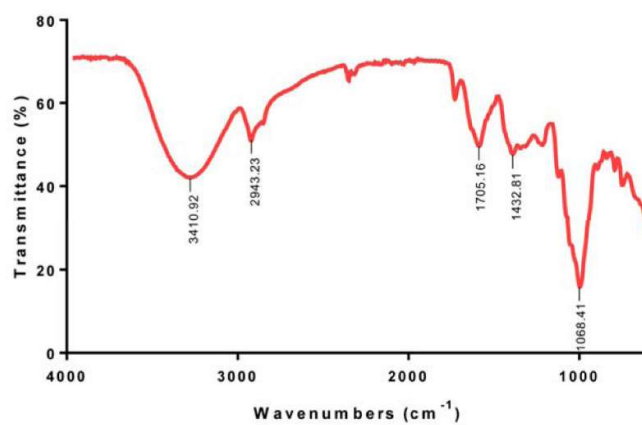

Figure S1. The Fourier transform infrared analysis of CTPS in ranges of 4000-600 cm<sup>-1</sup>.

Supplement: Supplementary file 1 [file ijms-22-07512-s001.zip › ijms-1273670-supplementary.pdf]
